# Attachment of Histidine Tags to Recombinant Tumor Necrosis Factor-Alpha Drastically Changes Its Properties

**DOI:** 10.1100/tsw.2002.215

**Published:** 2002-05-15

**Authors:** Irena Fonda, Maja Kenig, Vladka Gaberc-Porekar, Primo Pristovaek, Viktor Menart

**Affiliations:** National Institute of Chemistry, Hajdrihova 19, SI-1000, Ljubljana, Slovenia; Lek d.d., R&D, Celovška 135, Ljubljana, Slovenia

**Keywords:** IMAC, Immobilized Metal-ion Affinity Chromatography, histidine tags, His-tagged proteins, TNF-alpha, TNF, biological activity, inhibition of activity, purification, cleavage, cytotoxicity, truncated N-terminus, gyration radius, enterokinase, DAPase, steric hindrance, affinity

## Abstract

When studying two different histidine tags attached to the N-termini of the trimeric cytokine tumor necrosis factor alpha (TNF), the biological activity — measured as cytotoxicity on the L-929 cell line — of both tagged proteins was drastically reduced. The longer His10 tag reduced cytotoxicity to approximately 16% and the shorter His7 tag to 6% of the activity of their nontagged counterparts. After removal of the tags, biological activities reverted to the expected normal values, which clearly shows the key role of the attached histidine tags in diminishing biological activity. Studies on the mechanism of these effects revealed no specific interactions and showed that even the natural flexible N-terminus of TNF presents a steric hindrance for receptor binding, while any extension of the N-terminus increases this hindrance and consequently reduces biological activity. Also, in other proteins, the ligand or substrate binding sites may be hindered by histidine tags, leading to wrong conclusions about biological activity or other properties of the proteins. Thus caution is advised when using His-tagged proteins directly in screening procedures or in research.

### INTRODUCTION

In recent years, Immobilized Metal-Ion Affinity Chromatography (IMAC) techniques have become a popular tool for simple and effective isolation of recombinant proteins[1]. Although the first IMAC separations[2] were done with proteins containing naturally surface-exposed histidine residues, today this method uses primarily genetically engineered histidine clusters in the structure and especially histidine tags[3], which can be attached to either N- or C- termini of the target protein. As expression plasmids with sequences encoding for histidine tags are commercially available from many producers, histidine tagging and IMAC separations have become the method of choice in many research laboratories. Further, large-scale IMAC industrial separation of proteins is also promising, especially in combination with developing expanded bed adsorption technologies. In research, histidine tagging is often used for easy first-time isolations of newly expressed proteins, for rapid screening of mutant proteins, for structure-reactivity studies, etc. All studies with histidine-tagged proteins stem from the assumption and general belief that histidine tags do not affect protein folding, do not interfere with biological and physicochemical properties of the proteins, and do not elicit undesired immunological responses. This is certainly true in many cases, and even some examples of using histidine-tagged proteins directly for clinical purposes have been reported[4,5,6].

However, in the literature there are a few reports showing that histidine tags can alter protein properties. For example, C-terminal His6-tagged recombinant heparin factor II shows enhanced antithrombin and heparin cofactor activities as well as increased heparin-Sepharose binding compared to the wild-type protein, most probably attributable to the interaction between the N- and C-terminal parts of the tagged protein[7]. The fact that the enhanced activity is reversed after treatment with carboxypeptidase A clearly points to the key role of the His6 tag. The affect of a histidine tag on the activity of feline immunodeficiency virus integrase is quite complex[8]. Attachment of an His6 tag to its N-terminus significantly alters the selection of integration sites and increases the binding affinity to the DNA substrate, resulting in altered levels of catalytic activity. A hexahistidine tag-dependent protein dimerization has also been described[9], and lowered accumulation levels of histidine-tagged proteins have often been reported[10,11,12]. There are recent reports of totally altered properties of proteins, depending on the location of the His tag. For example, C-terminal fusions of His6 tag to the Mason-Pfizer Monkey Virus Capsid Protein induced the formation of organized thread- or sheet-like structures inside the *E. coli* cells[13]. In some cases, the reported specific activities for histidine-tagged proteins are higher than those of the wild-type counterparts[11], but most probably this can be ascribed to rapid and efficient one-step IMAC purification as compared to standard protein purification procedures involving approximately three purification steps. In contrast, hydantoinase-His6 was completely inactivated during elution from a metal affinity column[11]. In the case of DtpT (dipeptide and tripeptide transport) protein of *Lactococcus lactis,* the C-terminal addition of the Factor Xa cleavage site and hexahistidine tag reduced the biological activity to 50% of the wild-type activity[14]. Again, the normal activity was restored on removal of the His6 tag by treatment with Factor Xa.

These results indicate that histidine tagging can, to a greater or lesser extent, affect the properties of a protein. In a study on the mechanism of such affects, we report here an interesting example of two different histidine tags, attached to the N-termini of the trimeric cytokine TNF, which result in significantly reduced biological activity.

### MATERIALS AND METHODS

#### Chemicals

All chemicals used were of analytical grade and purchased from Sigma unless stated otherwise.

#### Histidine-Tagged Proteins

Two different histidine-tagged TNF proteins were used as model compounds. In both cases, histidine tags were genetically attached to the N-terminal part of TNF. His10-TNF bears a tag composed of 10 consecutive histidine residues and an enterokinase cleavage site, while in His7-(ΔN6)TNF the tag is attached to the truncated form of TNF lacking the first six amino acid residues. The His7 tag of His7-(ΔN6)TNF can be removed by dipeptidyl aminopeptidase (DAPase) digestion. The composition of both tags is shown in Table 1.

#### Cloning and Expression

A synthetic TNF gene with *E. coli*-optimized codons was supplied by British Biotechnology. DNA sequences encoding respective histidine tags were added by inserting the gene into an appropriate expression plasmid. Plasmids and the bacterial strains used are shown in Table 2.

#### Production

The cells of transformed *E. coli* strains were grown in shake flasks in LB medium containing 0.1 mg of ampicillin per ml, at 30°C and 160 rpm. After induction by isopropyl-1-thio-β-D-galactopyranoside (IPTG), 0.5 mM final concentration, cultures were grown until early stationary phase and then collected by centrifugation at 5000 rpm.

**TABLE 1**
**Composition of the N-Terminal Part of Model Proteins His10-TNF and His7-(ΔN6)TNF**

| Protein | Tag sequence | Cleavage enzyme |
|---|---|---|
| His10-TNF | MG(H)$_{10}$SSGHIDDDDK**↓**HM**VRSSSRTPSD…(TNF)** | Enterokinase |
| His7-(ΔN6)TNF | MK↓HH↓HH↓HH↓HM↓HA**↓TPSD…(TNF)** | DAPase |

Legend: ↓ DAPase cleavage site; **↓** putative final cleavage site; amino acid residues belonging to TNF are in bold.

**TABLE 2**
**Expression Plasmids and Bacterial Strains**

| Construct | Expression plasmid | Bacterial strain |
|---|---|---|
| His10-TNF | pET-19b (Novagen) | *E. coli* BL21(DE3) |
| His7-(ΔN6)TNF | TAGZyme pQE-2 (Qiagen) | *E. coli* DH5α or *E. coli* BL21(DE3) |

### Purification of Histidine-Tagged TNF Proteins

Bacterial pellets were resuspended in 50 mM TRIS/HCl, 30 mM NaCl, pH 8.0, and homogenized with an EmulsiFlex-C5 (Avestin) homogenizer. Nucleic acids were removed by precipitation with 0.1% polyethyleneimine. After centrifugation at 15,000 rpm, the supernatant containing histidine-tagged TNF proteins was precipitated at 65% saturated ammonium sulfate. Aliquots of the precipitate were stored at 4°C until chromatographic separation.

### IMAC Isolation of Histidine-Tagged TNF Proteins

All chromatographic procedures were carried out using a Knauer HPLC system equipped with two HPLC pumps 64 (Knauer), variable UV-Vis wavelength monitor (Knauer), and fraction collector FRAC-100 (Amersham Pharmacia Biotech).

His10-TNF was isolated on a 2-ml column HR 10/2 (Amersham Pharmacia Biotech) packed with Co-TALON Metal Affinity Resin (Clontech). The ammonium sulfate precipitate containing His10-TNF was dissolved in 50 mM K-phosphate, 0.5 M NaCl, pH 7.2, and applied to the column in the same buffer. After stringent washing with imidazole and N-lauroyl sarcosine, His10-TNF was eluted with 50 mM EDTA. The resulting His10-TNF was over 99% pure as determined by densitometric analysis of Coomassie-stained SDS-PAGE gel.

His7-(ΔN6)TNF was isolated on an HR 10/2 column (Amersham Pharmacia Biotech) packed with 2 ml Ni-NTA Superflow (Qiagen). The ammonium sulfate precipitate containing His7-(ΔN6)TNF was dissolved in 50 mM K-phosphate, 0.5 M NaCl, pH 7.2 buffer containing 5 mM imidazole and 0.5% N-lauroyl sarcosine and applied to the column in the same buffer. After washing with 50 mM imidazole containing buffer, His7-(ΔN6)TNF was eluted with 50 mM EDTA. Over 95% pure His7-(ΔN6)TNF was obtained as determined by densitometric analysis of Coomassie-stained SDS-PAGE gel.

Protein concentration was determined by the Bradford procedure[15].

SDS-PAGE was carried out in the vertical electrophoresis system Mini-Protean III (Bio-Rad) using 15% separating gels with 1 cm 4% stacking gel.

Densitometric purity evaluation of the isolated proteins was performed on Imaging Densitometer GS-670 (Bio-Rad) using Molecular Analyst software.

### Removal of Histidine Tags

His10-TNF was cleaved by EKMax enterokinase (Invitrogen). His10-TNF and enzyme were mixed in the mass ratio 100:1, $Ca^{2+}$ ions were added, and the mixture incubated at room temperature for 16 h.

His7-(ΔN6)TNF was cleaved by DAPase (Qiagen) following the protocol from TAGZyme Handbook for exoproteolytic cleavage of N-terminal His tags. Then 320 mU of DAPase was used to digest 1 mg of His7-(ΔN6)TNF at 37°C for 48 h to achieve 95% removal of His7 tags. Unprocessed and partially cleaved His7-(ΔN6)TNF, histidine-tagged DAPase, and cleaved-off His7 tags were removed on Ni-NTA Superflow (Qiagen). (ΔN6)TNF appeared in the flow-through fractions while all the contaminants were retained.

Adequacy of N-termini of pure proteins was confirmed by N-terminal amino acid sequencing on a Procise system 492A (PE Applied Biosystems).

### Biological Activity of TNF Proteins

Biological activity was measured as cytotoxic activity against the L-929 cell line according to the procedure of Flick and Gifford[16]. The procedure included seeding 2 x $10^4$ cells in 100 µl culture medium into 96-well microtitre plates and incubating for 24 h (37°C, 5% $CO_2$). Serial dilutions of internal TNF standard (calibrated to the NIBSC TNF standard 87/650) and TNF derivatives were added to the wells in the presence of 2 µg/ml actinomycin D. After incubation at 37°C, 5% $CO_2$ for 20 h, viable cells were fixed with 2.5% glutaraldehyde and stained with 0.5% crystal violet in 20% methanol. After solubilizing the cells in 1% SDS, the optical density was measured at 570 nm. The potency of the TNF proteins was determined by comparing the dilution yielding 50% of maximal cytotoxicity to the dilution of an internal standard TNF, yielding 50% of maximal cytotoxicity.

### Influence of Synthetic PHis7 Oligo-Peptide

The PHis7 oligopeptide MKHHHHHHHMHA was synthesized by Pepscan Systems (Lelystad, Netherlands) and was more than 95% pure.

For studying the effect of the attached His7 tag on the biological activity, mixtures of truncated (ΔN6)TNF and PHis7 were prepared in various molar ratios. PHis7 peptide was dissolved in Milli-Q water at a concentration of 10 mg/ml. Then 10x, 100x, 1000x, and 5000x molar excess of the peptide was added to the protein and the biological activity determined.

### Models of His-Tagged TNF Proteins

For basic modeling and generation of His-tagged protein pictures, a Swiss-PDBViewer v3.7b2 was used. Tags were added in alpha-helical arrangement, since its length is close to the average end-to-end distance. In modeling of the tags, we avoided the use of extended chains because they present a vastly exaggerated view of the available space; a statistically more sound view is presented by the average end-to-end distance that, however, cannot be modeled soundly. We emphasize that no preferred secondary structure of the tag is presumed.

## RESULTS AND DISCUSSION

### Expression level and accumulation as soluble protein and/or as inclusion bodies

Transcription and translation rates are probably not significantly affected by His-tagging at the N-terminus of the protein, as both histidine codons are used with similar frequencies. In *E. coli* expression levels exceeding 30% of total proteins are usually achieved using strong promoters like T7.

The accumulation levels of His-tagged proteins have been reported to be reduced in comparison to the levels of wild-type proteins[10,11,12,17]. However, these data have not been systematically collected and analyzed. Presumably, reduced accumulation levels of His-tagged proteins can in some cases be attributed to increased sensitivity of the tagged proteins to proteolytic attack[17].

Two facts are evident when expressing His-tagged TNF proteins with the intention of producing as much of the target protein as possible. First, reduced accumulation level, as already

**TABLE 3**
**Accumulation of His-Tagged TNF Proteins**

| Protein | *E. coli* strain | % of His-tagged protein in the cytoplasm* | % of His-tagged protein in the insoluble fraction** |
|---|---|---|---|
| His10-TNF | BL21(DE3) | 13 | 0 |
| His7-(ΔN6)TNF | BL21(DE3) | 34 | 33 |
| His7-(ΔN6)TNF | DH5α | 31 | nd |

*Soluble His-tagged proteins were almost quantitatively extracted from ammonium sulfate precipitates by using IMAC.
**The nature of His-tagged proteins in inclusion bodies was not determined.

**TABLE 4**
**Composition and Polarity/Hydrophobicity of the N-Terminal Parts of His10-TNF and His7-(ΔN6)TNF***

| Protein | Amino acid sequence of flexible part of N-terminus |
|---|---|
| His10-TNF | MGHHHHHHHHHHSSGHIDDDDKHMVRSSSRTPSDK-compactTNF |
| His7-(ΔN6)TNF | MKHHHHHHHMHATPSDK-compactTNF |

*Histidines and hydrophobic amino acid residues are marked in yellow, while hydrophilic (charged and polar) residues are marked in blue.

mentioned, can be attributed to proteolytically susceptible bonds present on the exposed and flexible N-terminus, which contains basic amino acid residues. This is probably the explanation for the low accumulation of His10-TNF (Table 3).

Second, many His-tagged proteins appear to be more inclined to form inclusion bodies. Our results show that the stretch of consecutive histidines is mildly hydrophobic. It would not be expected, however, that attaching a His-tag to a highly hydrophilic protein would reduce protein solubility in vivo. On the other hand, a combination of a mildly hydrophobic His-tag and an already partially hydrophobic protein can result in reduced solubility as well as formation of inclusion bodies. This is well-illustrated in the case of His7-(ΔN6)TNF (Table 3), in which the proteolytically susceptible region was excised and His7 tag attached to the truncated form (Table 4). The resulting His-tagged N-terminal region has decreased hydrophilicity, which will influence the solubility of the protein in vivo as well as in vitro.

An alternative explanation for the reduced accumulation of His-tagged proteins could be interference of His-tags with the folding process. Misfolded His-tagged proteins can be rapidly degraded by proteolysis or these proteins can accumulate as insoluble inclusion bodies. This would equally well explain the above-mentioned observations.

### Hydrophobicity of His-Tagged Proteins *In Vitro*

The 3D structure of TNF shows that the first six amino acid residues ($V^1RSSSR^6$) are completely flexible and exposed, since they are not visible in the X-ray structure[18]. The next five amino acid residues ($T^7PSDK^{11}$) are also at least partially flexible, as deletion studies up to the 10th amino acid did not destroy biological activity[19]. However, removal of 12 amino acid residues completely destroys biological activity, probably because the compact, biologically active structure is not formed.

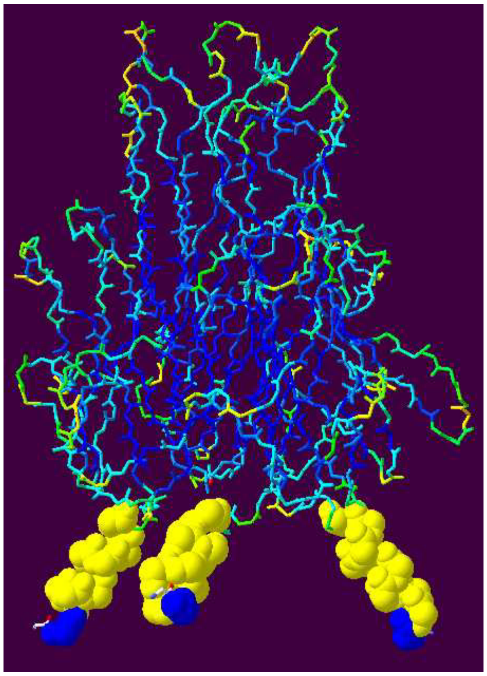

FIGURE 1A

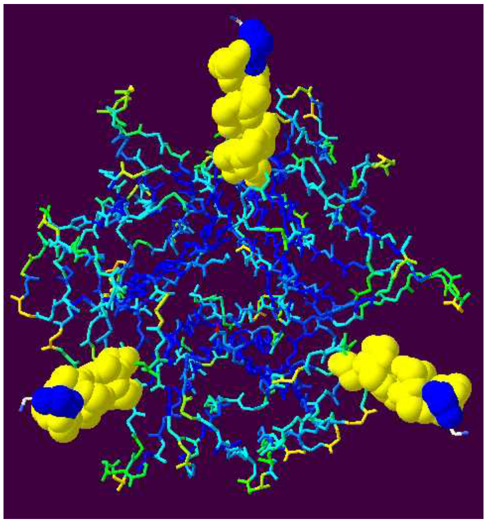

FIGURE 1B

**FIGURE 1.** Model of His7-(ΔN6)TNF. (A) Side view. (B) Bottom view. Tags are colored as in Table 4. Histidines and hydrophobic amino acid residues are in yellow, while hydrophilic (charged and polar) residues are in blue. Protein backbone is colored by accessibility: blue (interior) and green/yellow (surface).

In TNF, being a trimer, the amino acid composition of the exposed and symmetrically, triangularly arranged affinity tags (Fig. 1 and Fig. 2) could be expected to have some influence on the solubility of the protein.

This is supported by some observations during protein purification. Especially in the case of His7-(ΔN6)TNF, protein hydrophobicity was significantly increased. For example, during ultrafil-

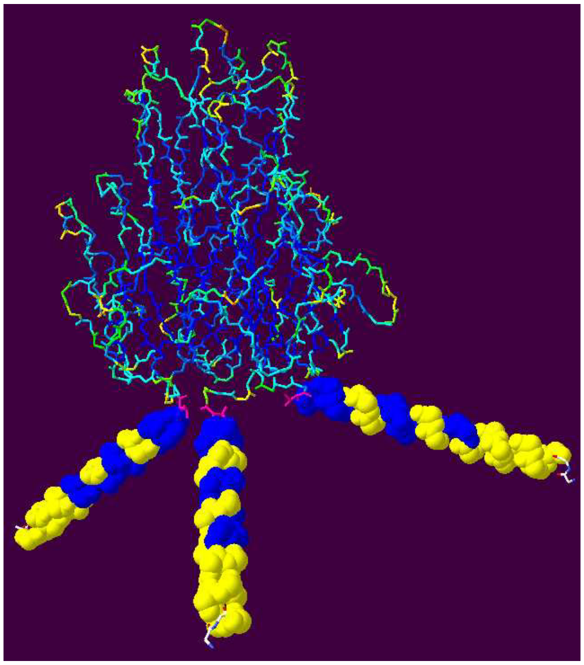

**FIGURE 2A**

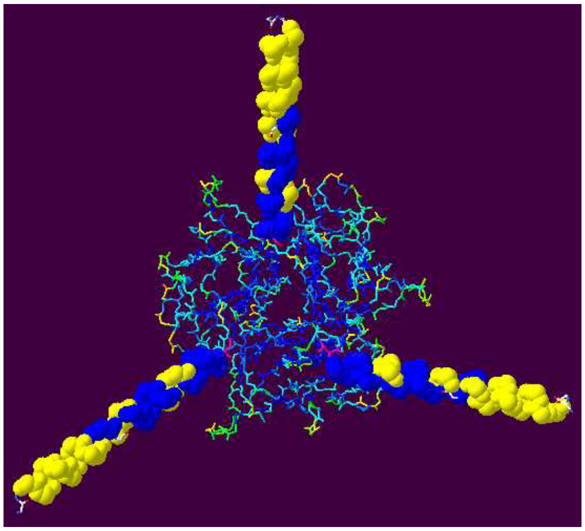

**FIGURE 2B**

**FIGURE 2.** Model of His10-TNF. (A) Side view. (B) Bottom view. Tags are colored as in Table 4. Histidines and hydrophobic amino acid residues are in yellow, while hydrophilic (charged and polar) residues are in blue. Protein backbone is colored by accessibility: blue (interior) and green/yellow (surface).

tration on Amicon membrane YM10, more than half the His7-(ΔN6)TNF was precipitated on the membrane and on the walls of the plastic housing of the ultrafiltration cell, even though the total concentration of the protein never exceeded 1 mg/ml. The solubility of natural TNF is higher than 10 mg/ml. It appears that His7-(ΔN6)TNF is also more prone to damage by freezing to -20°C (for storage), which results in loss of soluble material and a consequent drop in biological

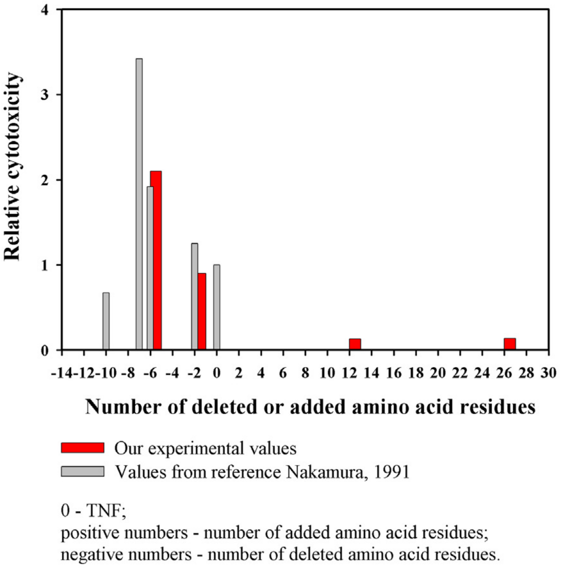

**FIGURE 3**. Relative cytotoxicity of truncated and His-tagged forms of TNF-alpha.

activity. Hydrophobic tags somehow assist denaturation, perhaps by increasing interaction with hydrophobic walls of plastic vials and membranes. It is known that TNF can rapidly lose biological activity, especially at concentrations below 1 μg/ml if the vials were not previously coated by a protective protein, usually albumin (unpublished observations). As native TNF alone is able to adhere to the vessel walls and denature there, it is reasonable to conclude that hydrophobic tags assist this process. In the case of His7-(ΔN6)TNF, the first six amino acid residues of TNF - charged and very polar - are deleted (Table 4). Removal of this polar part of the N-terminus and replacement by the slightly hydrophobic stretch of His-tag results in considerably increased hydrophobicity of the trimeric TNF as a whole. With His10-TNF, we have not observed any drastic changes regarding solubility and cold- or wall-induced denaturation, which is consistent with the more hydrophilic nature of its N-terminus.

### Biological Activity (Cytotoxicity) of Truncated and His-Tagged Forms of TNF

By successive deletions of 2 to 12 N-terminal residues of TNF, it was shown that the cytotoxicity of the truncated forms changes (Fig. 3), reaching the highest value after deletion of the first seven amino acid residues[19,20,21] . It was concluded that the N-terminal sequence of TNF plays an important role in modulating biological activity[19]. However, in the present context, it appears that the flexible and extended N-terminus is merely a prerequisite for cleavage of the membrane form of TNF by TACE (TNF-alpha-converting enzyme). To our knowledge, there is no evidence that nature uses truncated forms to regulate TNF biological activity. On the other hand, new TNF analogs have been designed for cancer therapy using truncated forms and the addition of basic amino acid residues to achieve increased specific antitumor activity and reduced systemic toxicity[22,23]. Thus, the flexible N-terminus can also be considered as a relict without definite regulative or other biological function. It appears that the native N-terminus itself interferes only sterically with TNF receptor binding and that no specific affinity interaction is involved. The extent of this influence can be roughly described by the radius of gyration[24], assuming that the N-terminus is completely flexible, resembling random coil motion. The addition of new amino

**TABLE 5**
**Biological Activity of His-Tagged TNF Proteins and Their Forms After Tag Removal**

| Protein | Cytotoxicity (IU/mg) | Biological activity (%) |
|---|---|---|
| (ΔN2)-TNF* | $2.7 \times 10^7$ | 100% |
| His10-TNF | $4.3 \times 10^6$ | 16% |
| (ΔN6)-TNF | $6.2 \times 10^7$ | 100% |
| His7-(ΔN6)TNF | $4.0 \times 10^6$ | 6.5% |

*Truncated TNF form obtained from His10-TNF after enterokinase cleavage. This is unspecific cleavage resulting in mixture of 80% (ΔN2)-TNF and 20% (ΔN6)-TNF (as judged from the N-terminal amino acid sequence determination).

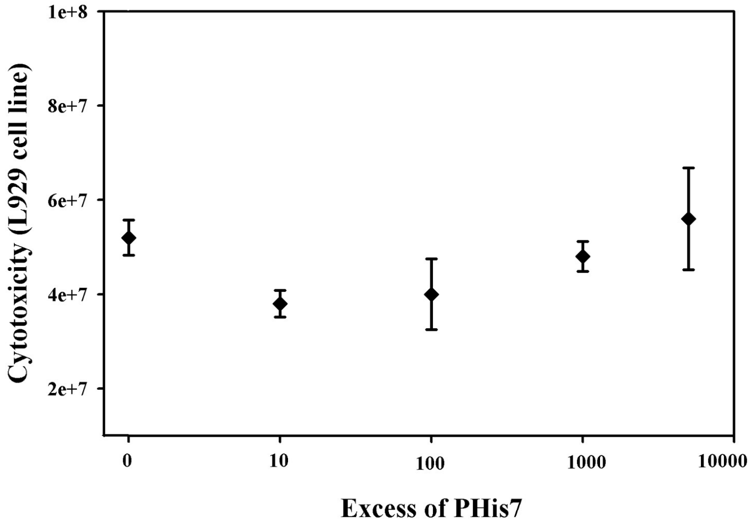

**FIGURE 4**. Influence of excess PHis7 peptide on cytotoxicity.

acid residues (e.g., histidine tags) to the native N-terminus would presumably increase interference and consequently diminish biological activity. The cytotoxicity of our His-tagged TNF proteins confirms this assumption (Table 5 and Fig. 3). Unexpectedly, we found that the shorter His7 tag reduced cytotoxicity to the same extent as the much longer His10 tag. One possible reason could be specific interaction of His7 tag with the receptor binding site. This was checked by competition study with an excess of synthetic peptide as described below.

### Competition Experiment using Synthetic Oligo-peptide PHis7

As a direct measure of the interaction between the receptor binding site and an His-tag attached to the N-terminus located nearby, we determined the influence of excess free PHis7 oligo-peptide.

In Fig. 4, it is shown that even up to $5 \times 10^3$ molar excess, PHis7 has no inhibitory effect on specific cytotoxicity of the truncated form (ΔN6)-TNF, as determined on L-929 cells. We conclude

**TABLE 6**
**Biological Activity in Relation to the Calculated $R_{ave}$**

| Protein | Ratio: specific activity TNF analog/specific activity TNF | Average end-to-end distance ($R_{ave}$) in Å | Reference |
|---|---|---|---|
| His10TNF | 0.14 | 32.2 | this study |
| (ΔN2)TNF | 0.9 | 16.2 | this study |
| His7(ΔN6)TNF | 0.13 | 22.4 | this study |
| (ΔN6)TNF | 2.1 | 11.9 | this study |
| TNF* | 1 | 18.0 | |
| (ΔN2)TNF | 1.25 | 16.2 | [19] |
| (ΔN6)TNF | 1.92 | 11.9 | [19] |
| (ΔN7)TNF | 3.42 | 10.5 | [19] |
| (ΔN10)TNF | 0.67 | 3.5 | [19] |
| (ΔN12)TNF | 0 | 0 | [19] |

*For comparison of values from two studies (Nakamura et al.[19] and ours), the specific activity of natural TNF was taken as 3 x $10^7$ IU/mg.

that the free peptide does not bind at or near the TNF receptor binding site, whether the His7 tag is attached to the N-terminus or not. At the moment, we do not have enough experimental data to deduce any reasonable explanation. However, the failure of the free peptide to interfere with binding of (ΔN6)TNF to the TNF receptor could also be interpreted in terms of the lack of context or adequate preorganization of the soluble and mobile short peptide in comparison to the bound one.

In general, we may expect to find other cases of interference of His-tags with protein biological activity (for example, in proteins containing hydrophobic clefts for substrate or ligand binding).

### Effect of the N-terminal Random Coil Chain

It appears that the native N-terminus itself interferes only sterically with TNF receptor binding and that no specific attractive interaction is involved. The extent of this influence can be roughly described by the radius of gyration of the structurally undefined N-terminal chain or even more conveniently by its average end-to-end distance $R_{ave}$[24]. In the present case, this would be the average distance between the N-terminal residue and a residue of the protein that does not possess a well-defined structure but is attached to a structurally well-defined residue at its C-terminus. For the latter, we chose the D10 residue because the N-terminus including D10 can be removed from TNF without deleting its biological activity (see (ΔN10)TNF in Table 6). $R_{ave}$ then signifies the average distance between the N-terminal residue and the K11 residue. We have calculated $R_{ave}$ for some TNF analogs already described[19] and for some of our own TNF derivatives (Table 6) using the formula

$$<R_{ave}^2> = 2\, N\, L^2\, (N + 2) / (N + 1),[24,25]$$

where N is the number of residues in the random coil chain and L is the distance between two sequential C$\alpha$ atoms (3.85 Å).

Fig. 5 shows the dependence of the activity (expressed as the ratio between the specific activities of the TNF analog and the wild type) on the calculated $R_{ave}$. The trend indicates that the activity has a maximum at $R_{ave}$ = 10.5 Å and drops almost to zero for larger values. At the moment,

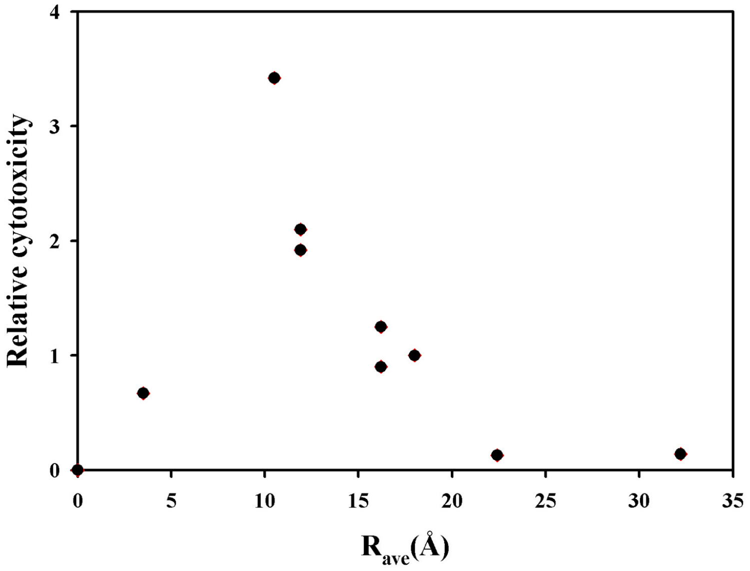

**FIGURE 5**. Relation between relative biological activity and calculated $R_{ave}$.

the amount of data is not sufficient for a full explanation of this behavior. It seems, however, that the natural TNF ($R_{ave}$ = 18 Å), which already contains a flexible N-terminus, presents a steric hindrance to the receptor; the hindrance is reduced on deletion of N-terminal residues, the highest activity being observed with (ΔN7)-TNF. More than 10 deletions are deleterious for the activity. On the other hand, any prolongation of the N-terminus relative to the wild-type TNF (e.g., with His-tags) reduces the activity.

### CONCLUSIONS

When studying two different histidine tags attached to the N-terminal parts of the trimeric TNF molecule, several interesting properties of the histidine-tagged proteins were revealed. In the case of His10-TNF, the accumulation level of the tagged protein was significantly lower than that of the untagged protein expressed under the same conditions. In contrast, the accumulation level of His7-(ΔN6)TNF was over 30% of total protein but its solubility was lower, and part of the protein appeared in the form of insoluble inclusion bodies, which is not the case with native TNF expressed in *E. coli*. Most probably the reason lies in the hydrophobic nature of the new N-terminal part of the protein. Obviously, a stretch of seven mildly hydrophobic histidine residues in combination with the truncated form of TNF lacking six amino acid residues (five of them hydrophilic) made a molecule less hydrophilic and influenced its solubility properties in vivo as well as in vitro. Most interestingly, both histidine-tagged proteins, His10-TNF and His7-(ΔN6)TNF, exhibited significantly reduced biological activities, which reverted to the expected normal values after removal of the tags. This clearly shows the key role of the attached histidine tags in changing protein biological properties.

On the basis of our results, alteration of the properties of proteins as a result of the addition of histidine tags can be expected in other cases. This sends an important message that it can be misleading to draw conclusions about function or activity of newly expressed proteins based on their histidine-tagged counterparts alone.

### ACKNOWLEDGMENTS

We acknowledge Mrs. J. Lenarčič for laboratory preparation of plasmid constructs, Mrs. A. Jesenko and Mrs. T. Preradov-Vasle for protein analyses, and Mrs. Š. Peternel for early chromatographic experiments with His7-(ΔN6)TNF. We also wish to thank Prof. R. Pain for reading the manuscript and for helpful discussions.

This work was supported by the Ministry of Education, Science and Sport of Slovenia.

## References

1. Arnold, F.H. (1991) Metal-affinity separations: a new dimension in protein processing. *Bio/Technology* **9**, 150–155.
2. Porath, J., Carlsson, J., Olsson, I., and Belfrage, G. (1975) Metal chelate affinity chromatography, a new approach to protein fractionation. *Nature* **258**, 598–599.
3. Hochuli, E., Bannwarth, W., Doebeli, H., Gentz, R., and Stueber, D. (1988) Genetic approach to facilitate purification of recombinant proteins with a novel metal chelate adsorbent. *Bio/Technology* **[vol. 6?]** 1321–1325.
4. Kaslow, D.C. and Shiloach, J. (1994) Production, purification and immunogenicity of a malaria transmission-blocking vaccine candidate: TBV25H expressed in yeast and purified using nickel-NTA agarose. *Bio/Technology* **12**, 494–499.
5. Takacs, B.J. and Girard, M.F. (1991) Preparation of clinical grade proteins produced by recombinant DNA technologies. *J. Immunol. Methods* **143**, 231–240.
6. Casey, J.L., Keep, P.A., Chester, K.A., Robson, L., Hawkins, R.E., and Begent, R.H. (1995) Purification of bacterially expressed single chain Fv antibodies for clinical applications using metal chelate chromatography. *J. Immunol. Methods* **179**, 105–116.
7. Bauman, S.J. and Church, F.C. (1999) Enhancement of heparin cofactor II anticoagulant activity. *J. Biol. Chem.* **274**, 34,556–34,565.
8. Shibagaki, Y., Holmes, M.L., Appa, R.S., and Chow, S.A. (1997) Characterization of feline immunodeficiency virus integrase and analysis of functional domains. *Virology* **230**, 1–10.
9. Wu, J. and Filutowicz, M. (1999) Hexahistidine (His6)-tag dependent protein dimerization: a cautionary tale. *Acta Biochim. Pol.* **46**, 591–599.
10. Muller, S., Adomeit, A., Kaufmann, R., Appelhans, H., Passow, H., Reissmann, S., and Liebmann, C. (2000) Expression and functional characterization of a pHis-tagged human bradykinin B2 receptor in COS-7 cells. *Biol. Chem.* **381**, 343–347.
11. Pietzsch, M., Wiese, A., Ragnitz, K., Wilms, B., Altenbuchner, J., Mattes, R., and Syldatk, C. (2000) Purification of recombinant hydantoinase and L-N-carbamoylase from Arthrobacter aurescens expressed in Escherichia coli: comparison of wild-type and genetically modified proteins. *J. Chromatogr. B Biomed. Sci. Appl.* **737**, 179–186.
12. Gaberc-Porekar, V. and Menart, V. (2001) Perspectives of immobilized-metal affinity chromatography. *J. Biochem. Biophys. Methods* **49**, 335–360.
13. Rumlova, M., Benedikova, J., Cubinkova, R., Pichova, I., and Ruml, T. (2001) Comparison of classical and affinity purification techniques of Mason-Pfizer monkey virus capsid protein: the alteration of the product by an affinity tag. *Protein Express. Purif.* **23**, 75–83.
14. Hagting, A., Knol, J., Hasemeier, B., Streutker, M.R., Fang, G., Poolman, B., and Konings, W.N. (1997) Amplified expression, purification and functional reconstitution of the dipeptide and tripeptide transport protein of Lactococcus lactis. *Eur. J. Biochem.* **247**, 581–587.
15. Bradford, M.M. (1976) A rapid and sensitive method for the quantitation of microgram quantities of protein utilizing the principle of protein-dye binding. *Anal. Biochem.* **72**, 248–254.
16. Flick, D.A. and Gifford, G.E. (1984) Comparison of in vitro cell cytotoxic assays for tumor necrosis factor. *J. Immunol. Methods* **68**, 167–175.
17. Gaberc-Porekar, P., Menart, V., Jevševar, S., Videnšek, A., and Štalc, A. (1999) Histidines in affinity tags and surface clusters for immobilized metal-ion affinity chromatography of trimeric tumor necrosis factor alpha. *J. Chromatogr. A* **852**, 117–128.
18. Eck, M.J. and Sprang, S.R. (1989) The structure of tumor necrosis factor-alpha at 2.6 A resolution: Implications for receptor binding. *J. Biol. Chem.* **264**, 17,595-17,605.
19. Nakamura, S., Masegi, T., Fukuoka, M., Kitai, K., Kato, A., Ichikawa, Y., Watanabe, N., and Niitsu, Y. (1991) Interactions between novel tumor-necrosis-factor-alpha mutants and receptors on tumor and normal-cells. *Agricultural and Biological Chemistry* **55**, 53–57.

20. Creasey, A.A., Doyle, L.V., Reynolds, M.T., Jung, T., Lin, L.S., and Vitt, C.R. (1987) Biological effects of recombinant human tumor necrosis factor and its novel muteins on tumor and normal cell lines. *Cancer Res.* **47**, 145–149.
21. Sidhu, R.S. and Bollon, A.P. (1989) Tumor necrosis factor analogs: identification of functional domains. *Anticancer Res.* **9**, 1569–1576.
22. Soma, G., Tsuji, Y., Tanabe, Y., Noguchi, K., Kitahara-Tanabe, N., Gatanaga, T., Inagawa, H., Kawakami, M., and Mizuno, D. (1988) Biological activities of novel recombinant tumor necrosis factor having N-terminal amino acid sequences derived from cytotoxic factors produced by THP-1 cells. *J. Biol. Response Mod.* **7**, 587–595.
23. Nakamoto, T., Inagawa, H., Takagi, K., and Soma, G. (2000) A new method of antitumor therapy with a high dose of TNF perfusion for unresectable liver tumors. *Anticancer Res.* **20**, 4087–4096.
24. Creighton, T.E. (1993) *Proteins: Structures and Molecular Properties*. W.H. Freeman and Company, New York, 1993.
25. Atkins, P.W. (1987) *Physical Chemistry*. Oxford University Press, Oxford, England.
